# Cancer screening utilization among insured beneficiaries in Saudi Arabia, 2023–2024: a national analysis of trends, calendar variation, and distribution across breast, cervical, and colorectal screening programs

**DOI:** 10.3389/fonc.2026.1837321

**Published:** 2026-08-28

**Authors:** Abdurahman Alloghbi

**Affiliations:** 1 Department of Oncology, College of Medicine, King Khalid University, Abha, Saudi Arabia; 2 Department of Oncology, Saudi German Hospital, Khamis Mushait, Saudi Arabia

**Keywords:** breast cancer, cancer screening, cervical cancer, colorectal cancer, health insurance, health services research, preventive care, Saudi Arabia

## Abstract

**Background:**

Breast, cervical, and colorectal cancer (CRC) screening uptake in Saudi Arabia has been reported as consistently low, but no national longitudinal description of insurance-linked screening utilization has been published. This study examined temporal trends, calendar variation, and demographic distribution of cancer screening among privately insured beneficiaries in Saudi Arabia.

**Methods:**

We analyzed six publicly available Council of Health Insurance datasets reporting monthly breast, cervical, and colorectal cancer screening counts among insured beneficiaries during 2023–2024 (24 monthly observations per cancer type). Because the datasets contain aggregate counts without enrollment or eligibility denominators, only absolute utilization counts could be analyzed. Two log-linear regression models were fitted to each monthly series: an unadjusted time-trend model and a primary adjusted model incorporating Ramadan and an October–December 2024 step-change term. Sensitivity analyses evaluated three alternative Ramadan exposure definitions.

**Results:**

Total screening counts increased by 56.3%, from 102,048 in 2023 to 159,496 in 2024. In the adjusted model, statistically significant upward monthly trends were observed for breast (+3.0%; 95% CI 1.5–4.5), cervical (+2.7%; 95% CI 1.6–3.8), and CRC screening (+4.6%; 95% CI 3.3–5.9) (all *p* < 0.001). April screening counts were 32.4%–41.6% below model-predicted values across all programs, consistent with a Ramadan–Eid-associated reduction in elective screening activity and robust across all sensitivity analyses. A significant CRC-specific step change was identified during October–December 2024 (+46.6%; 95% CI 13.1–89.9; *p* = 0.006), whereas no comparable effect was observed for breast or cervical screening. CRC screening consistently showed male predominance, with male-to-female count ratios of 1.53 in 2023 and 1.57 in 2024.

**Conclusions:**

Insurance-linked cancer screening utilization increased substantially across all three screening programs between 2023 and 2024. A reproducible Ramadan–Eid-associated decline and a CRC-specific late-2024 increase were identified, providing insights relevant to screening program planning and evaluation. Because eligible-population denominators were unavailable, these findings describe utilization counts rather than screening participation or coverage and highlight the need for denominator linkage to enable future assessment of coverage and equity.

## 1 Introduction

Saudi Arabia faces a growing cancer burden alongside sweeping health system reforms that have placed prevention and early detection at the center of national health policy. Breast cancer is the most common malignancy among Saudi women, with a substantial increase in annual incidence documented over the past two decades (1). Colorectal cancer (CRC) represents the largest contributor to total cancer incidence across both sexes, accounting for 14.4% of all diagnosed cancers in 2020, with Riyadh reporting the highest age-standardized incidence rates nationally (2, 3). Cervical cancer has lower absolute incidence relative to these two cancers, yet high human papillomavirus (HPV) positivity among cervical cancer cases underscores the importance of cytological surveillance in the Saudi context (4).

Despite this burden, population-level cancer screening uptake has been documented as consistently low in Saudi Arabia across all three cancer types. Among women aged 40–75 years, mammography uptake was reported at 4.9% in a cross-Gulf comparative study (5). Colorectal cancer screening has been described as alarmingly low, with reported rates of 4.2% for any CRC screening in one study and under 10% for age-appropriate screening in another, with fewer than 1% of older adults having undergone colonoscopy (6, 7). Cervical cancer screening is similarly limited, with only 33.4% of surveyed individuals reporting ever having had a Pap smear (8). Documented barriers include personal fear, limited knowledge, low provider awareness, and insufficient infrastructure for endoscopy-based follow-up (9, 10).

Saudi Arabia’s Health Sector Transformation Program under Vision 2030 has reported population health coverage reaching 97.4%, anchored by the Cooperative Health Insurance framework that mandates private-sector employee enrollment and specifies preventive care as a covered benefit domain (11, 12). The updated Essential Benefit Package (EBP), effective October 2022, designates preventive services in primary care with low cost-sharing (0–5%, up to 25 SAR) and covers early detection examinations and diagnostics for breast cancer with a maximum benefit limit of 500,000 SAR (13, 14). Enrollment under the CHI program grew from approximately 4.8 million workers in 2008 to over 12 million by 2017 (15). This structural expansion makes a national description of insurance-linked screening utilization timely. It is important to state at the outset, however, that the present study cannot assess the effect of the EBP or of coverage expansion on screening activity: the available datasets begin in January 2023, after the October 2022 activation of the updated EBP, and no pre-policy observations exist against which a comparison could be made.

Saudi Arabia lacks a truly population-based screening model for breast and colorectal cancer, and organized program reports have been limited to single-center or regional settings (16–18). The Saudi Cancer Registry has documented limitations in linkage to mortality statistics and primary care records, constraining rigorous impact evaluation (19). No prior study has used national administrative insurance data to characterize the utilization of all three major screening programs simultaneously over time.

The Council of Health Insurance recently released publicly accessible administrative datasets reporting cancer screening activity among CHI beneficiaries across 2023 and 2024, stratified by cancer type, month, age group, and sex. These datasets provide, for the first time, a national-level longitudinal window into insurance-linked cancer screening utilization in Saudi Arabia. Accordingly, the objectives of this study are threefold: (1) to quantify the trend in monthly screening counts across the 24-month observation window for the breast, cervical, and colorectal programs; (2) to characterize and formally test within-year calendar variation in screening counts, with particular attention to the Ramadan–Eid period and to October awareness-month activity; and (3) to describe the distribution of screening counts across age groups and, for CRC, across sexes. Because the available data contain no eligible-population denominators, these objectives are framed in terms of absolute utilization counts; the study does not aim to estimate screening participation, population coverage, or the effect of any policy intervention.

## 2 Materials and methods

### 2.1 Study design

This is a retrospective analysis of an aggregated monthly administrative time series, repeated cross-sectional in structure, assembled from publicly available aggregate data. No individual-level data were accessed. Reporting follows the Strengthening the Reporting of Observational Studies in Epidemiology (STROBE) guidelines.

### 2.2 Data source

Six datasets were downloaded from the Saudi open data portal (open.data.gov.sa), released by the Council of Health Insurance of Saudi Arabia. The datasets cover three cancer screening programs—breast, cervical, and colorectal—for the calendar years 2023 and 2024, and are described in **Table 1**. All datasets report the monthly number of CHI-insured female beneficiaries (or all-sex beneficiaries for CRC) who underwent screening at healthcare providers accredited by the CHI across the Kingdom of Saudi Arabia.

**Table 1:** Description of CHI cancer screening datasets used in this study.

Dataset	Year	Cancer type	Population	Age groups	Stratification
Breast Cancer Screening 2023	2023	Breast	CHI female beneficiaries	40–49, 50–69	Age group × Month
Breast Cancer Screening 2024	2024	Breast	CHI female beneficiaries	40–49, 50–69	Age group × Month
Cervical Cancer Screening 2023	2023	Cervical	CHI female beneficiaries	21–29, 30–65	Age group × Month
Cervical Cancer Screening 2024	2024	Cervical	CHI female beneficiaries	21–29, 30–65	Age group × Month
Colorectal Cancer Screening 2023	2023	Colorectal	CHI beneficiaries (all sexes)	45–49, 50–75	Sex × Age group × Month
Colorectal Cancer Screening 2024	2024	Colorectal	CHI beneficiaries (all sexes)	45–49, 50–75	Sex × Age group × Month

CHI, Council of Health Insurance. All datasets are pre-aggregated monthly counts and contain no enrollment or eligibility information.

It is important to characterize the granularity of these data precisely. The datasets are pre-aggregated monthly counts published by cancer type, month, age band, and (for CRC) sex. They are not claims-level records, they are not linked to any other administrative file, and they contain no enrollment, eligibility, or beneficiary-level information. No variables permitting identification of repeat screening, screening interval, test result, or downstream diagnostic follow-up are available.

Breast cancer screening data report the number of female CHI beneficiaries who underwent mammography, disaggregated by age group (40–49 years; 50–69 years) and month. Cervical cancer screening data report the number of female CHI beneficiaries who underwent cervical cancer screening (Pap smear or equivalent cytological test), disaggregated by age group (21–29 years; 30–65 years) and month. Colorectal cancer screening data report the number of CHI beneficiaries of both sexes who underwent CRC screening, disaggregated by sex (female, male), age group (45–49 years; 50–75 years), and month. Age groups in these datasets are consistent with national and international guideline-recommended screening ages for each cancer type.

### 2.3 Variables and measures

The primary outcome variable was the monthly count of CHI beneficiaries who underwent cancer screening (screen_count), as reported in each dataset. Aggregate variables derived for analysis included: (1) annual totals per cancer type and year, computed by summing monthly counts for the “All” age group and, for CRC, the “All” sex category; (2) year-over-year absolute and relative change from 2023 to 2024; (3) age-group-specific annual totals and their proportional contribution to annual totals; (4) sex-specific annual totals and male-to-female (M:F) count ratios for CRC, stratified by age group; (5) quarterly totals (Q1: January–March; Q2: April–June; Q3: July–September; Q4: October–December); and (6) monthly totals for identification of peak and trough screening months.

No eligible-population denominators are published in these datasets, and none were available to the author. Consequently no participation rate, coverage level, or standardized uptake ratio could be computed, and all outcomes reported here are absolute counts of screening events among CHI-insured beneficiaries. The M:F quantity reported for CRC is therefore a ratio of counts, not a ratio of utilization rates.

### 2.4 Statistical analysis

Analyses comprised two components: (i) descriptive enumeration of counts, proportions, ratios, and percentage changes; and (ii) inferential log-linear regression of the 24-month monthly series (January 2023 through December 2024) to estimate the secular trend, the Ramadan-period calendar effect, and the October–December 2024 step change.

#### 2.4.1 Descriptive analysis

Counts, proportions, and percentage changes were calculated. Year-over-year percentage change was computed as: ((2024 count − 2023 count)/2023 count) × 100. M:F count ratios were computed by dividing the total male screening count by the total female screening count, overall and by age group. Proportional age-group contributions were calculated as the age-specific count divided by the total annual count for the applicable cancer type and year. Peak and trough months were identified across cancer types and years, and quarterly distributions were expressed as a proportion of the annual total. Monthly seasonal indices were computed as the ratio of each month’s average count (averaged across both years) to the overall monthly grand mean, separately for each cancer type; indices above 1.0 indicate above-average activity for that calendar month. All computations were performed in Python 3 using the pandas library (version 3.12.3). Figures were produced using matplotlib (version 3.10).

#### 2.4.2 Regression analysis

Two models were fitted to each cancer type’s monthly count series by ordinary least squares (OLS) on the natural log-transformed outcome, where t is a sequential monthly time index (t = 1 for January 2023 through t = 24 for December 2024):

- Model 1 (unadjusted time trend): ln(count) = β_0_ + β_1_·t + ϵ
- Model 2 (adjusted, primary specification): ln(count) = β_0_ + β_1_·t + β_2_·Ramadan + β_3_·PostOct2024 + ϵ

where Ramadan is a binary indicator coded 1 for April 2023 (t = 4) and April 2024 (t = 16) and 0 otherwise, and PostOct2024 is a binary indicator coded 1 for October, November, and December 2024 (t = 22, 23, 24) and 0 otherwise.

**Figure 1:**
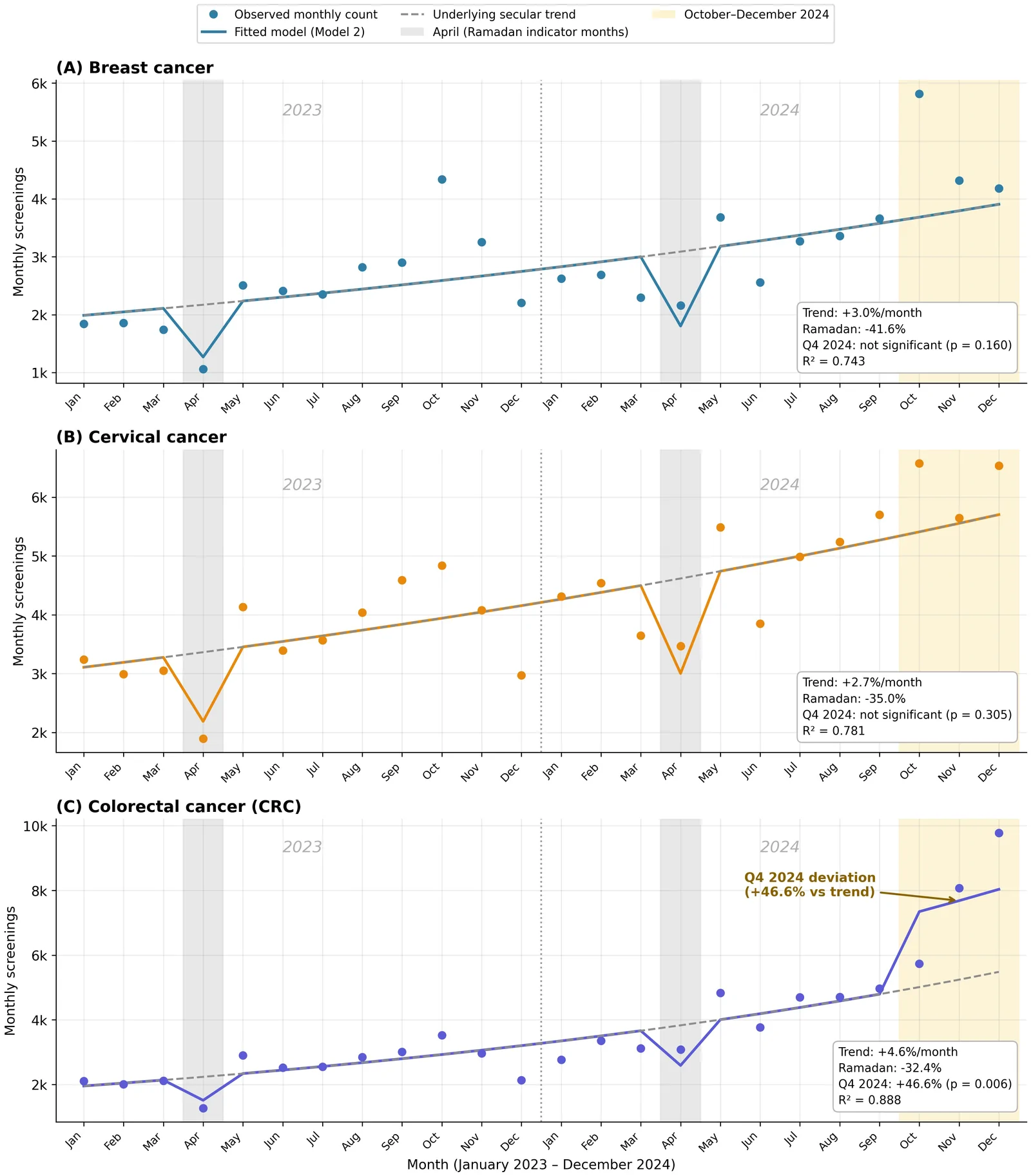
Log-linear time trend and step-change analysis of monthly cancer screening counts, January 2023 through December 2024 (n = 24 months). **(A)** Breast cancer screening. **(B)** Cervical cancer screening. **(C)** Colorectal cancer (CRC) screening. Circles represent observed monthly counts. Solid colored lines represent the fitted adjusted model, Model 2 (ln[count] = β_0_ + β_1_·time + β_2_·Ramadan + β_3_·PostOct2024). Dashed grey lines represent the underlying secular time trend with the Ramadan and step-change terms set to zero, illustrating the counterfactual trajectory absent those effects. Grey shaded bands indicate the April months coded by the Ramadan indicator. Yellow shaded band indicates October–December 2024. The fitted step is drawn only where the post-October 2024 term is statistically significant (CRC); for the breast and cervical programs the term was not significant and the fitted line is shown without a step, with the p value annotated. Inset annotation boxes report the Model 2 monthly trend rate (+3.0%, +2.7%, and +4.6% for breast, cervical, and CRC respectively), the Ramadan estimate (−41.6%, −35.0%, −32.4%), and the model R² (0.743, 0.781, 0.888), matching **Table 2**. Vertical dotted line marks the transition between 2023 and 2024. CHI, Council of Health Insurance; CRC, colorectal cancer.

Model 2 was designated the primary specification, because it is the model from which the calendar and step-change estimates are derived and the model displayed in **Figure 1**; reporting the trend estimate from the same model that generates the other two headline estimates avoids any mixing of estimates across specifications. All estimates quoted in the Abstract, Results, Discussion, and Conclusion are Model 2 estimates unless explicitly labelled as Model 1. Model 1 estimates are reported in **Table 2** for transparency and comparison.

**Table 2:** Log-linear regression results for monthly cancer screening counts, January 2023 – December 2024 (n = 24 months per program).

Model/parameter	Breast β (95% CI)	Breast p	Cervical β (95% CI)	Cervical p	CRC β (95% CI)	CRC p
Model 1 — unadjusted time trend						
Intercept	7.445 (7.223 to 7.666)	<0.001	7.931 (7.765 to 8.097)	<0.001	7.397 (7.201 to 7.593)	<0.001
Time (per month)	0.038 (0.022 to 0.053)	<0.001	0.031 (0.020 to 0.043)	<0.001	0.057 (0.043 to 0.071)	<0.001
— as % change per month	+3.8 (2.2 to 5.5)		+3.2 (2.0 to 4.4)		+5.9 (4.4 to 7.3)	
R²	0.535		0.589		0.771	
Adjusted R²	0.514		0.570		0.761	
Model 2 — adjusted (primary)						
Intercept	7.566 (7.375 to 7.756)	<0.001	8.015 (7.875 to 8.155)	<0.001	7.534 (7.376 to 7.692)	<0.001
Time (per month)	0.029 (0.015 to 0.044)	<0.001	0.026 (0.016 to 0.037)	<0.001	0.045 (0.032 to 0.057)	<0.001
— as % change per month	+3.0 (1.5 to 4.5)		+2.7 (1.6 to 3.8)		+4.6 (3.3 to 5.9)	
Ramadan (April)	−0.538 (−0.845 to −0.230)	0.002	−0.430 (−0.656 to −0.204)	<0.001	−0.391 (−0.646 to −0.136)	0.005
— as % deviation	−41.6 (−57.0 to −20.6)		−35.0 (−48.1 to −18.5)		−32.4 (−47.6 to −12.7)	
Post-October 2024	0.218 (−0.094 to 0.530)	0.160	0.116 (−0.114 to 0.345)	0.305	0.382 (0.123 to 0.641)	0.006
— as % deviation	+24.3 (−9.0 to +69.8)		+12.3 (−10.7 to +41.2)		+46.6 (+13.1 to +89.9)	
R²	0.743		0.781		0.888	
Adjusted R²	0.704		0.748		0.872	
Durbin–Watson	1.49		2.13		1.71	

Coefficients are OLS estimates on the natural log of monthly counts. Percentage quantities are (exp[β] − 1) × 100, with limits obtained by transforming the corresponding coefficient confidence limits. Model 1: ln(count) = β_0_ + β_1_·t. Model 2 (primary): ln(count) = β_0_ + β_1_·t + β_2_·Ramadan + β_3_·PostOct2024. All estimates quoted in the Abstract, Results, and Discussion are Model 2 estimates unless labelled otherwise. CRC, colorectal cancer.

Coefficients were estimated by OLS, with standard errors computed from the variance-covariance matrix of the residuals and two-sided t-tests used to evaluate significance against H_0_: β_i_ = 0. The exponentiated slope coefficient, (exp[β_1_] − 1) × 100, was interpreted as the estimated monthly percentage change in screening counts; the Ramadan coefficient as the percentage deviation from the expected trend in April months, (exp[β_2_] − 1) × 100; and the step-change coefficient as the excess above the secular trend attributable to October–December 2024, (exp[β_3_] − 1) × 100. Ninety-five percent confidence intervals are reported for all coefficients, with limits for derived percentage quantities obtained by transforming the corresponding coefficient confidence limits. Model fit was assessed using R², adjusted R², and the Akaike information criterion (AIC); residual autocorrelation was assessed using the Durbin–Watson statistic. All regression analyses were performed in Python 3 using NumPy (version 2.4), SciPy (version 1.17), and statsmodels.

#### 2.4.3 Sensitivity analysis of the Ramadan exposure definition

In the Saudi civil calendar, Ramadan spanned 23 March to 20 April 2023 (Eid al-Fitr 21 April 2023) and 11 March to 9 April 2024 (Eid al-Fitr 10 April 2024). Ramadan therefore falls across two calendar months in each year and in unequal proportions between years: April 2023 contained 20 Ramadan days, whereas April 2024 contained only nine, with the majority of the 2024 Ramadan falling in March. A single April indicator may consequently misclassify exposure. Three alternative exposure definitions were therefore substituted for the April indicator in Model 2, with all other terms unchanged:

- S1: a binary March–April indicator (both months, both years);
- S2: a continuous variable equal to the proportion of each month’s calendar days falling within Ramadan;
- S3: a continuous variable equal to the proportion of each month’s calendar days falling within Ramadan or the Eid al-Fitr holiday window, defined as the day of Eid plus three days, to capture the post-Ramadan holiday closure of elective services.

For the continuous specifications the coefficient represents the deviation associated with a full month of exposure and is therefore not directly comparable in magnitude with the binary specifications.

Log-linear OLS was selected over Poisson or negative binomial regression as a pragmatic choice: with only 24 monthly observations per cancer type and four model parameters, the residual degrees of freedom (df = 20) are insufficient to reliably estimate the overdispersion parameter required by negative binomial regression, and OLS on log-transformed outcomes provides interpretable, approximately unbiased coefficient estimates under this constraint. All regression findings are based on a short 24-month series and should be treated as indicative of directionality and magnitude rather than as definitive estimates; replication with longer series as additional annual CHI data become available is strongly encouraged.

### 2.5 Ethics statement

This study used publicly available, de-identified aggregate administrative data with no access to individual-level patient records. In accordance with the Declaration of Helsinki and local regulations, ethical approval was not required for secondary analysis of publicly available aggregate data. No personal data were collected or processed.

## 3 Results

### 3.1 Overall screening trends

All results below describe absolute screening counts among CHI-insured beneficiaries, not population-based rates; no denominators were available and no coverage or participation quantity is reported.

**Figure 2:**
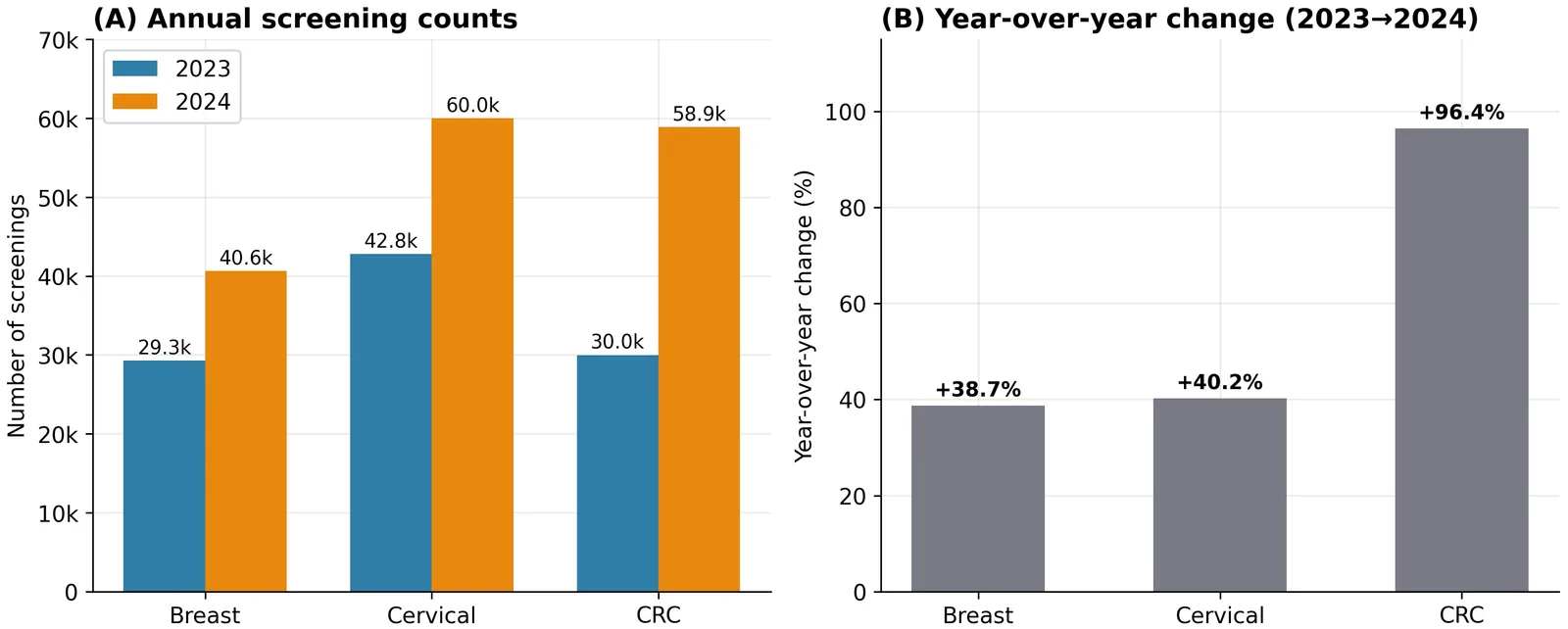
Annual cancer screening counts and year-over-year change among CHI-insured beneficiaries, 2023–2024. **(A)** Total screening counts by cancer type and year. **(B)** Year-over-year percentage change from 2023 to 2024. CHI, Council of Health Insurance; CRC, colorectal cancer.

Total cancer screening counts across all three programs increased by 56.3%, from 102,048 in 2023 to 159,496 in 2024. Year-over-year growth was observed in all three programs, with substantial variation in magnitude across cancer types (**Figure 2**; **Table 3**). CRC screening showed the greatest absolute and relative increase, nearly doubling over the two-year period, while breast and cervical screening counts grew at comparable rates.

**Table 3:** Annual cancer screening counts, age-group breakdown, and year-over-year changes among CHI beneficiaries (2023–2024).

Variable	2023	2024	Change (n)	Change (%)
Breast cancer screening				
Annual total (all ages)	29,284	40,616	+11,332	+38.7%
Age 40–49 years	15,446	21,555	+6,109	+39.6%
Age 50–69 years	13,838	19,061	+5,223	+37.7%
Cervical cancer screening				
Annual total (all ages)	42,779	59,985	+17,206	+40.2%
Age 21–29 years	7,775	11,082	+3,307	+42.5%
Age 30–65 years	35,004	48,903	+13,899	+39.7%
Colorectal cancer screening				
Annual total (all sexes/ages)	29,985	58,895	+28,910	+96.4%
Female — total	11,841	22,924	+11,083	+93.6%
Female — age 45–49	2,185	4,222	+2,037	+93.2%
Female — age 50–75	9,656	18,702	+9,046	+93.7%
Male — total	18,144	35,971	+17,827	+98.2%
Male — age 45–49	3,920	8,078	+4,158	+106.1%
Male — age 50–75	14,224	27,893	+13,669	+96.1%
M:F count ratio — overall	1.53	1.57	—	—
M:F count ratio — age 45–49	1.79	1.91	—	—
M:F count ratio — age 50–75	1.47	1.49	—	—
Combined (all 3 programs)				
Total annual screenings	102,048	159,496	+57,448	+56.3%

M:F, male-to-female. M:F values are ratios of counts, not of utilization rates. CHI, Council of Health Insurance.

**Table 4:** Monthly screening counts by cancer type and year, 2023 and 2024.

Month	Jan	Feb	Mar	Apr	May	Jun	Jul	Aug	Sep	Oct	Nov	Dec	Annual total
Breast 2023	1,840	1,856	1,743	1,059	2,508	2,411	2,352	2,819	2,899	**4,340**	3,253	2,204	**29,284**
Breast 2024	2,624	2,691	2,294	2,162	3,683	2,558	3,268	3,362	3,660	**5,814**	4,319	4,181	**40,616**
Cervical 2023	3,237	2,991	3,052	1,894	4,131	3,395	3,565	4,036	4,590	**4,839**	4,076	2,973	**42,779**
Cervical 2024	4,312	4,541	3,646	3,468	5,486	3,852	4,986	5,239	5,703	**6,575**	5,644	6,533	**59,985**
CRC 2023	2,112	2,014	2,120	1,272	2,901	2,525	2,551	2,851	3,011	**3,528**	2,962	2,138	**29,985**
CRC 2024	2,771	3,355	3,122	3,083	4,835	3,767	4,694	4,704	4,967	5,739	8,076	**9,782**	**58,895**

Peak monthly values for each cancer type and year are displayed in bold. CRC, colorectal cancer; CHI, Council of Health Insurance.

### 3.2 Breast cancer screening

**Figure 3:**
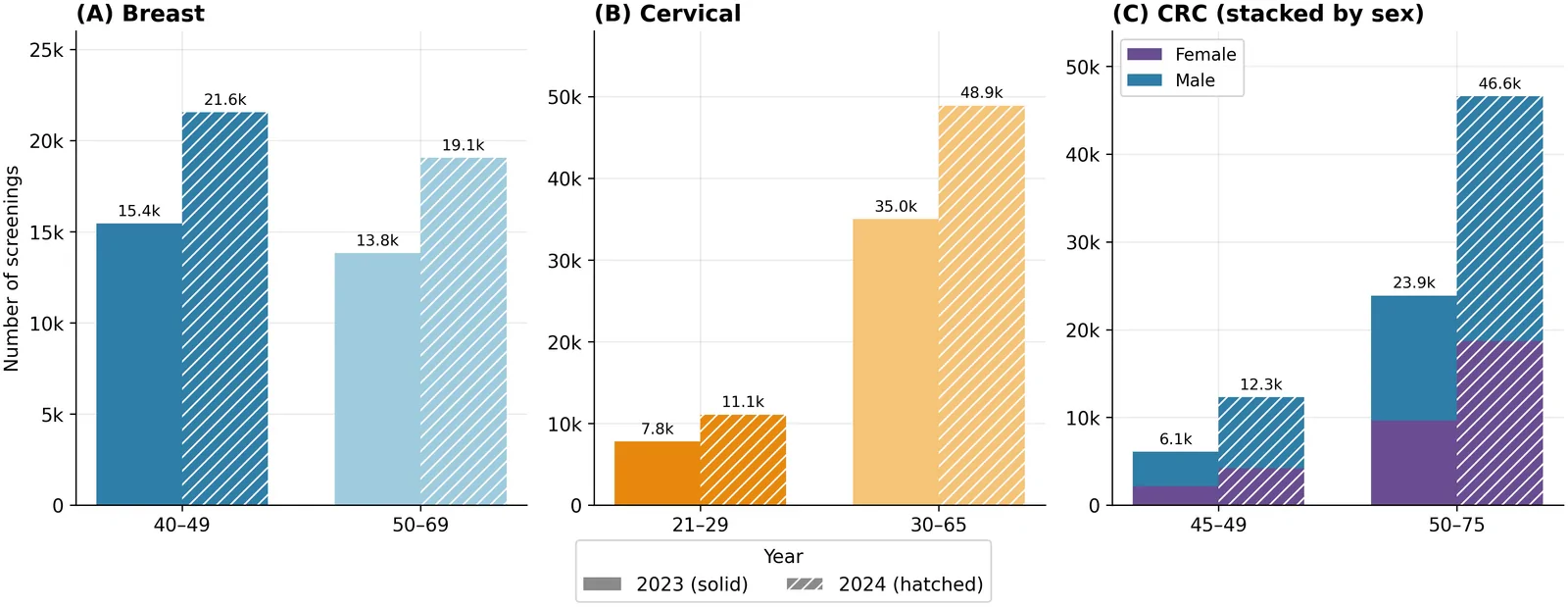
Age-group distribution of cancer screening counts by year. **(A)** Breast cancer screening by age group (40–49 and 50–69 years). **(B)** Cervical cancer screening by age group (21–29 and 30–65 years). **(C)** Colorectal cancer screening by age group (45–49 and 50–75 years), stratified by sex. Solid bars represent 2023; hatched bars represent 2024. In **(C)** bars are stacked by sex, with the total for each year labeled.

**Figure 4:**
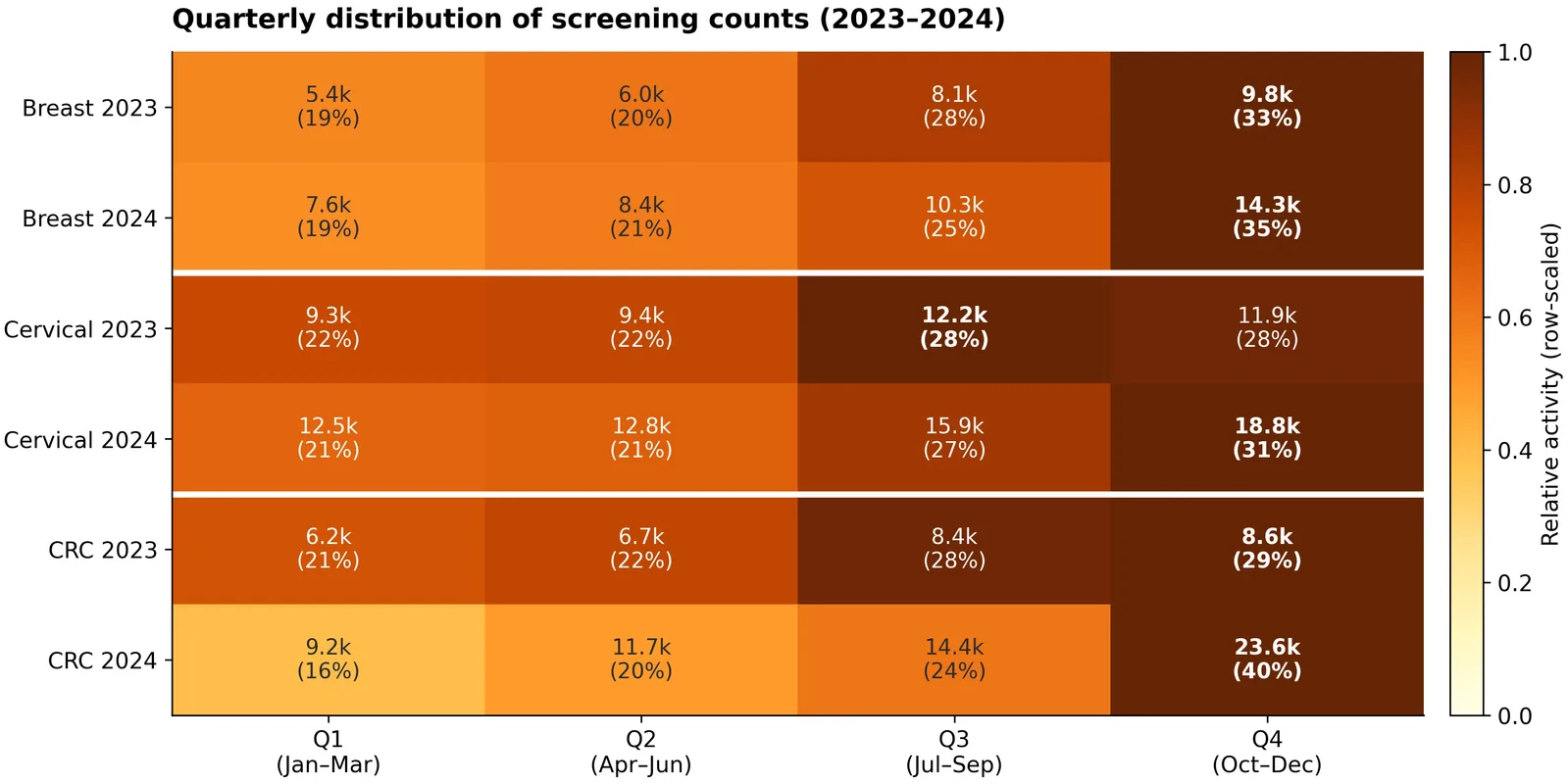
Quarterly distribution of cancer screening counts across all six CHI datasets (2023–2024). Cell values show quarterly screening count (thousands) and proportion of annual total. Color scale represents relative activity within each row (row-normalized). White horizontal lines separate cancer types. CHI, Council of Health Insurance; CRC, colorectal cancer.

The number of CHI female beneficiaries who underwent breast cancer screening (mammography) increased from 29,284 in 2023 to 40,616 in 2024, a year-over-year increase of 38.7% (absolute increase: 11,332). Growth was consistent across both age groups: counts among women aged 40–49 years increased from 15,446 to 21,555 (+39.6%), and among women aged 50–69 years from 13,838 to 19,061 (+37.7%). The proportional distribution between the two age groups remained essentially stable, with the 40–49 age group accounting for 52.7% of annual screenings in 2023 and 53.1% in 2024 (**Figure 3A**). Quarterly growth was consistent across the year, with the highest relative increase in Q4 (+46.1%, 9,797 to 14,314) (**Figure 4**).

### 3.3 Cervical cancer screening

The number of CHI female beneficiaries who underwent cervical cancer screening increased from 42,779 in 2023 to 59,985 in 2024, a year-over-year increase of 40.2% (absolute increase: 17,206). Cervical cancer screening was the highest-volume program in absolute terms in both years. Growth was distributed across both age strata: counts among women aged 21–29 years increased from 7,775 to 11,082 (+42.5%), and among women aged 30–65 years from 35,004 to 48,903 (+39.7%). The 30–65 age group consistently accounted for approximately 81.5–81.8% of annual cervical screenings across both years (**Figure 3B**). Quarterly analysis revealed the highest relative growth in Q4 (+57.7%) (**Figure 4**), driven notably by a marked increase in December 2024 (2,973 in December 2023 vs 6,533 in December 2024; +119.8%).

### 3.4 Colorectal cancer screening

The number of CHI beneficiaries who underwent CRC screening nearly doubled from 29,985 in 2023 to 58,895 in 2024, a year-over-year increase of 96.4% (absolute increase: 28,910). This growth rate substantially exceeded that of the breast and cervical programs. CRC screening counts increased across all sex and age subgroups: female beneficiaries aged 45–49 years from 2,185 to 4,222 (+93.2%), female beneficiaries aged 50–75 years from 9,656 to 18,702 (+93.7%), male beneficiaries aged 45–49 years from 3,920 to 8,078 (+106.1%), and male beneficiaries aged 50–75 years from 14,224 to 27,893 (+96.1%) (**Figure 3C**).

Quarterly analysis revealed that the exceptional growth in CRC screening was concentrated in Q4 2024. Q4 CRC screenings increased by 173.5% (8,628 in Q4–2023 to 23,597 in Q4 2024), compared with increases of 48.1%, 74.5%, and 70.7% in Q1–Q3, respectively. Specifically, November 2024 recorded 8,076 CRC screenings (compared with 2,962 in November 2023, + 172.7%), and December 2024 recorded 9,782 (compared with 2,138 in December 2023, + 357.5%). Together, November and December 2024 accounted for 30.3% of the entire 2024 CRC screening total, compared with 17.0% in 2023. This pattern was internally consistent across all sex and age subgroups and is examined further in §3.7 and §4.3.

### 3.5 Sex distribution of CRC screening counts

**Figure 5:**
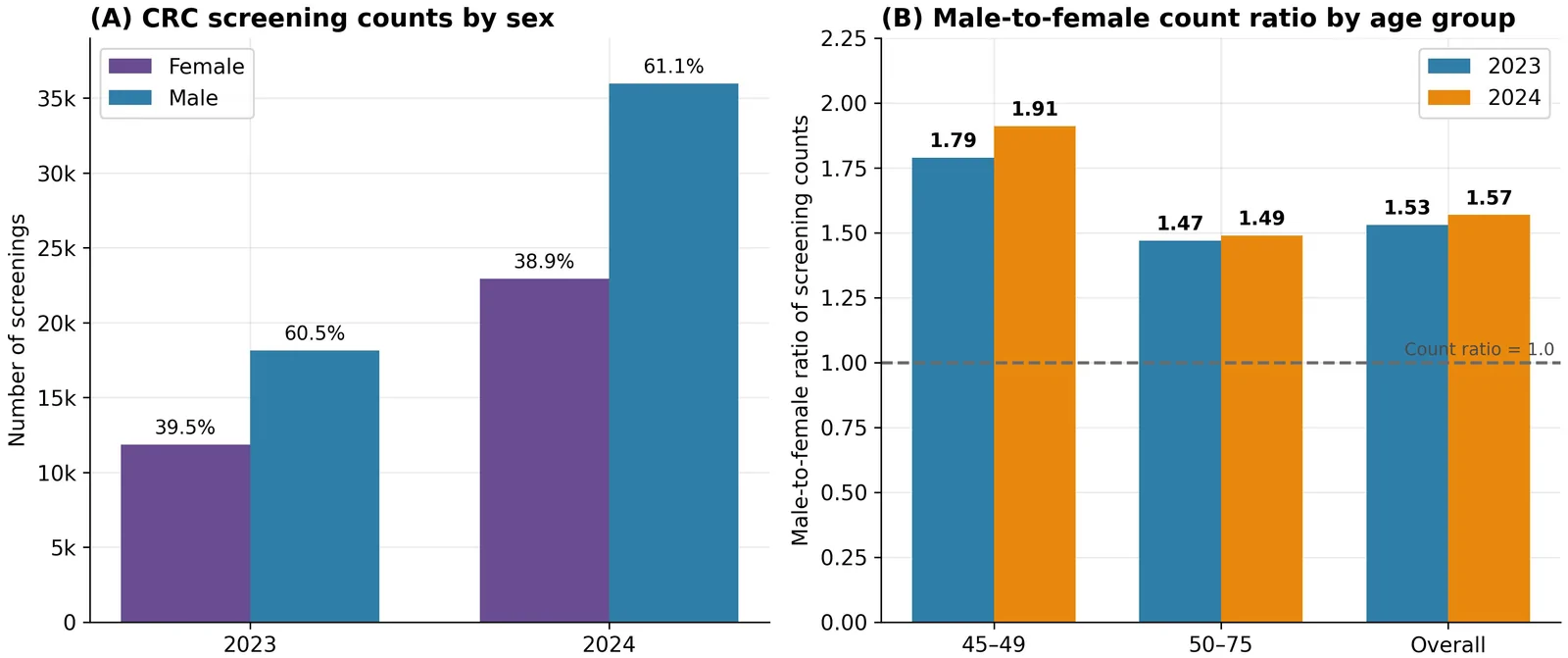
Distribution of colorectal cancer screening counts by sex among CHI-insured beneficiaries, 2023–2024. **(A)** Annual CRC screening counts by sex and year, with percentage share labeled. **(B)** Male-to-female ratio of screening counts (not rates) by age group and year. Dashed horizontal line indicates a count ratio of 1.0. CHI, Council of Health Insurance; CRC, colorectal cancer.

A consistent male predominance in CRC screening counts was observed across both years (**Figure 5**). Male beneficiaries accounted for 60.5% of total CRC screenings in 2023 (18,144 male vs 11,841 female) and 61.1% in 2024 (35,971 male vs 22,924 female). The M:F count ratio was 1.53 in 2023 and 1.57 in 2024. The male predominance was most pronounced in the younger age group (45–49 years), with a ratio of 1.79 in 2023 and 1.91 in 2024; in the older age group (50–75 years), the ratio was lower (1.47 in 2023 and 1.49 in 2024) but remained above parity (**Table 3**). As noted in §2.3, these are ratios of counts and not of utilization rates.

### 3.6 Seasonal and monthly patterns

**Figure 6:**
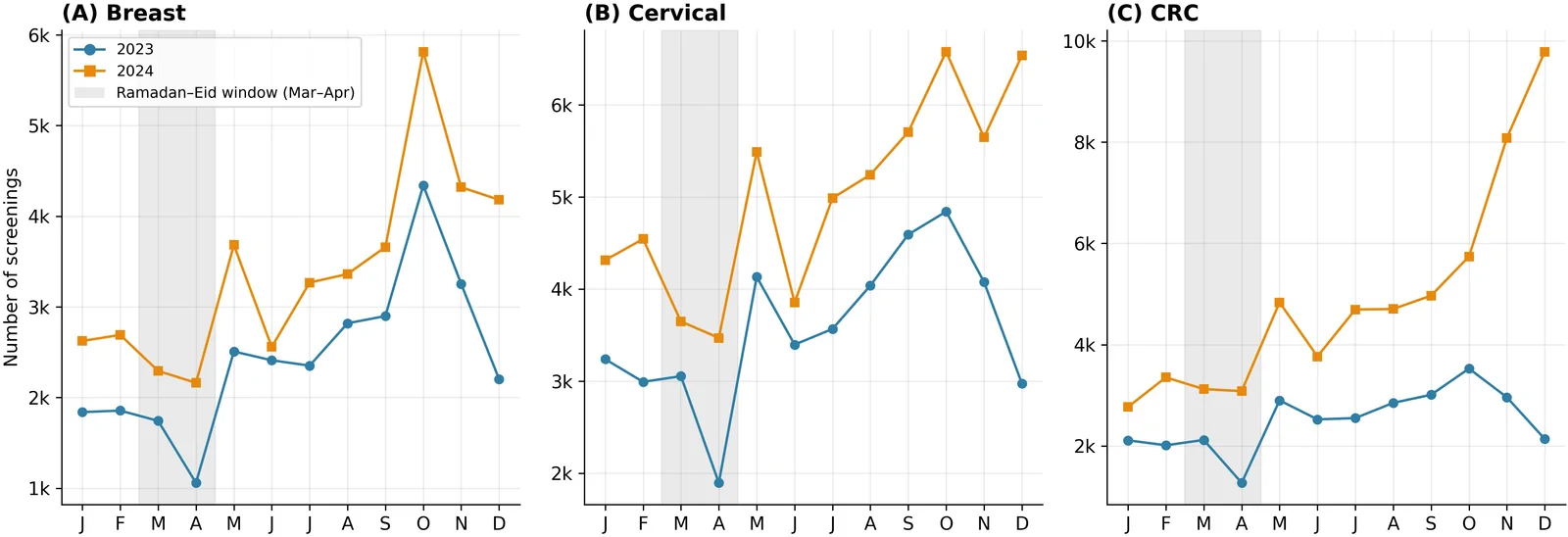
Monthly cancer screening counts among CHI-insured beneficiaries, 2023–2024. **(A)** Breast cancer screening. **(B)** Cervical cancer screening. **(C)** Colorectal cancer screening. Grey shaded band indicates the March–April window spanning the Ramadan–Eid period in both years (Ramadan 23 March – 20 April 2023 and 11 March – 9 April 2024; Eid al-Fitr 21 April 2023 and 10 April 2024). Note that the vertical scale differs between panels. CHI, Council of Health Insurance.

A consistent cross-cancer trough in April was identified across all three screening programs in both years (**Figure 6**). April recorded the lowest monthly screening counts for breast cancer (1,059 in 2023; 2,162 in 2024), cervical cancer (1,894 in 2023; 3,468 in 2024), and CRC (1,272 in 2023) in their respective years. This trough coincided with the Ramadan–Eid period in both years, although as noted in §2.4 the distribution of Ramadan days between March and April differed between 2023 and 2024. For 2024, CRC April was also among the lower months (3,083), though the year’s overall elevated volumes moderated the proportional dip compared with 2023.

October was the peak month for breast and cervical cancer screening in both years (breast: 4,340 in 2023; 5,814 in 2024; cervical: 4,839 in 2023; 6,575 in 2024), consistent with Breast Cancer Awareness Month programming. For CRC, October was the peak in 2023 (3,528) but was surpassed by November and December in 2024 as part of the Q4 deviation described above. The quarterly heatmap (**Figure 4**) summarizes relative activity distribution across all six datasets.

Seasonal index profiling revealed a consistent within-year pattern. October had the highest seasonal index for breast cancer (1.74), indicating October volumes on average 74% above the monthly grand mean. For cervical cancer the October index was 1.33; for CRC, the November (1.49) and December (1.61) indices reflect the Q4–2024 deviation. Across all three programs April had the lowest seasonal index (breast: 0.55; cervical: 0.63; CRC: 0.59).

### 3.7 Time trend, calendar, and step-change analysis

Regression results for both models are presented in **Table 2** and illustrated in **Figure 1**.

Model 1 (unadjusted time trend). A statistically significant upward monthly trend was identified for all three cancer types: breast +3.8% per month (β_1_ = 0.038, 95% CI 0.022 to 0.053; +3.8%, 95% CI 2.2 to 5.5; p < 0.001; R² = 0.535), cervical +3.2% per month (β_1_ = 0.031, 95% CI 0.020 to 0.043; +3.2%, 95% CI 2.0 to 4.4; p < 0.001; R² = 0.589), and CRC + 5.9% per month (β_1_ = 0.057, 95% CI 0.043 to 0.071; +5.9%, 95% CI 4.4 to 7.3; p < 0.001; R² = 0.771).
Model 2 (adjusted; primary specification). After adjustment for the Ramadan and post-October 2024 terms, the estimated monthly trend was +3.0% for breast cancer (β_1_ = 0.029, 95% CI 0.015 to 0.044; +3.0%, 95% CI 1.5 to 4.5; p < 0.001), +2.7% for cervical cancer (β_1_ = 0.026, 95% CI 0.016 to 0.037; +2.7%, 95% CI 1.6 to 3.8; p < 0.001), and +4.6% for CRC (β_1_ = 0.045, 95% CI 0.032 to 0.057; +4.6%, 95% CI 3.3 to 5.9; p < 0.001). These correspond to annualized increases of 42.2%, 37.3%, and 71.2% respectively. The trend was positive, statistically significant, and steepest for CRC under both specifications; the adjusted estimates are lower than the unadjusted estimates because the Ramadan and step-change terms absorb variance that the unadjusted trend would otherwise attribute to time.

The Ramadan indicator was statistically significant for all three cancer types. After accounting for the underlying time trend, April months were associated with a 41.6% reduction in breast cancer screening counts relative to the expected trend value (β_2_ = −0.538, 95% CI −0.845 to −0.230; −41.6%, 95% CI −57.0 to −20.6; p = 0.002), a 35.0% reduction for cervical cancer (β_2_ = −0.430, 95% CI −0.656 to −0.204; −35.0%, 95% CI −48.1 to −18.5; p < 0.001), and a 32.4% reduction for CRC (β_2_ = −0.391, 95% CI −0.646 to −0.136; −32.4%, 95% CI −47.6 to −12.7; p = 0.005).

The post-October 2024 step-change indicator was statistically significant for CRC (β_3_ = 0.382, 95% CI 0.123 to 0.641; +46.6%, 95% CI 13.1 to 89.9; p = 0.006), representing a 46.6% excess above the expected secular trend in October through December 2024. The same indicator was not statistically significant for breast cancer (β_3_ = 0.218, 95% CI −0.094 to 0.530; p = 0.160) or cervical cancer (β_3_ = 0.116, 95% CI −0.114 to 0.345; p = 0.305), indicating that the Q4–2024 deviation was specific to the CRC program.

Model 2 explained 74.3%, 78.1%, and 88.8% of the variance in log-transformed monthly counts for the breast, cervical, and CRC programs respectively (adjusted R² = 0.704, 0.748, 0.872). Durbin–Watson statistics were 1.49, 2.13, and 1.71, providing no strong evidence of residual autocorrelation, although with 24 observations these diagnostics have limited power.

### 3.8 Sensitivity of the Ramadan estimate to alternative exposure definitions

Results of the three alternative Ramadan exposure specifications are presented in **Table 5**. The estimated reduction was negative and statistically significant under every specification. Under the binary March–April indicator (S1) the estimated reductions were smaller than under the April-only definition (−33.9% breast, −26.2% cervical, −22.1% CRC), whereas the continuous proportion-of-days specifications yielded larger full-month-equivalent estimates (S2: −55.5%, −46.1%, −41.6%; S3: −52.9%, −43.9%, −39.8%). Model fit improved modestly under the continuous specifications, with S3 giving the lowest AIC for all three programs, consistent with exposure being genuinely fractional across two calendar months rather than confined to a single one.

**Table 5:** Sensitivity of the Ramadan estimate and the Q4–2024 CRC step change to alternative Ramadan exposure definitions.

Exposure definition	Breast: Ramadan effect (95% CI), p	Cervical: Ramadan effect (95% CI), p	CRC: Ramadan effect (95% CI), p	CRC: post-Oct 2024 effect (95% CI), p	AIC (breast/cervical/CRC)
Primary: April indicator	−41.6% (−57.0 to −20.6), 0.002	−35.0% (−48.1 to −18.5), <0.001	−32.4% (−47.6 to −12.7), 0.005	+46.6% (+13.1 to +89.9), 0.006	−6.0/−20.8/−14.9
S1: March–April indicator	−33.9% (−47.3 to −17.0), 0.001	−26.2% (−38.2 to −11.9), 0.002	−22.1% (−36.5 to −4.3), 0.020	+46.7% (+11.2 to +93.5), 0.009	−6.8/−18.7/−11.7
S2: proportion of Ramadan days	−55.5% (−70.8 to −32.2), <0.001	−46.1% (−60.8 to −25.7), <0.001	−41.6% (−59.6 to −15.4), 0.007	+46.4% (+12.5 to +90.5), 0.007	−8.0/−21.1/−14.1
S3: proportion of Ramadan + Eid days	−52.9% (−67.1 to −32.6), <0.001	−43.9% (−57.2 to −26.5), <0.001	−39.8% (−56.2 to −17.1), 0.003	+46.0% (+13.0 to +88.5), 0.006	−9.9/−23.4/−15.5

Each row substitutes the stated exposure variable for the April indicator in Model 2; the time trend and post-October 2024 terms are otherwise unchanged. For the continuous specifications (S2, S3) the estimate is the deviation associated with a full month of exposure and is not directly comparable in magnitude with the binary specifications. Ramadan dates (Saudi civil calendar): 23 March – 20 April 2023, Eid al-Fitr 21 April 2023; 11 March – 9 April 2024, Eid al-Fitr 10 April 2024. The Eid window in S3 is the day of Eid plus three days. AIC, Akaike information criterion (lower values indicate better fit).

The CRC post-October 2024 estimate was essentially invariant across all four Ramadan specifications (+46.6%, +46.7%, +46.4%, +46.0%; all p ≤ 0.009), indicating that this finding does not depend on how the Ramadan exposure is coded.

## 4 Discussion

This study provides the first national longitudinal description of cancer screening utilization among CHI-insured beneficiaries in Saudi Arabia, combining descriptive analysis with log-linear regression across 24 monthly observations for the breast, cervical, and colorectal programs (2023–2024). Four findings emerge. First, statistically significant upward monthly trends in screening counts were identified for all three cancer types, with CRC showing the steepest trajectory (+4.6% per month, 95% CI 3.3 to 5.9; R² = 0.888). Second, a quantified April trough was identified across all three programs, with counts 32.4% to 41.6% below model-predicted values, robust in direction and significance across four alternative Ramadan exposure definitions. Third, the Q4–2024 CRC deviation was statistically distinguishable from the background trend (β_3_ = 0.382, p = 0.006; +46.6%, 95% CI 13.1 to 89.9) and was cancer-specific, with no equivalent signal in the breast or cervical programs. Fourth, a persistent male predominance in CRC screening counts was documented, with M:F count ratios of 1.53 and 1.57 in 2023 and 2024.

Throughout the discussion below, it is essential to keep in view that these are counts of screening events without denominators. Growth in counts is not equivalent to growth in participation, and count ratios between subgroups are not equivalent to differences in uptake.

### 4.1 Trends and their interpretation

The estimated monthly trend rates from the primary adjusted model — +3.0% for breast, +2.7% for cervical, and +4.6% for CRC — correspond to annualized increases of 42.2%, 37.3%, and 71.2% and to doubling times of approximately 23.6, 26.3, and 15.5 months respectively. Model 2 explains 74% to 89% of the variance in monthly log-counts, indicating that time, the April calendar effect, and the Q4–2024 indicator together account for most of the observed month-to-month variability. Residual unexplained variance likely reflects provider-level fluctuation, undocumented outreach activity, and reporting lags inherent to administrative systems.

Two constraints limit what these trends can be taken to mean. First, published Saudi uptake estimates (4.9% for mammography; under 10% for CRC) are prevalence proportions computed against defined denominators and are therefore not commensurable with the utilization counts analyzed here (5–7); this study makes no inference about whether coverage has changed. Second, CHI enrollment itself expanded substantially over the period in which these data were collected (15), so some portion of the observed count growth may reflect a growing insured population rather than increased per-capita utilization. The two cannot be separated without enrollment denominators stratified by age, sex, and year.

The consistent positive slopes across all three programs are temporally coincident with the October 2022 activation of the updated Essential Benefit Package, which reinforced preventive care as a covered, low-cost-sharing benefit domain (13). We emphasize that this observation is a hypothesis and not a finding of this study. The available data begin in January 2023 and contain no pre-policy observations; formally attributing trend level or acceleration to the EBP would require a pre-post or interrupted-time-series design with an adequate pre-intervention window, which these datasets cannot support.

### 4.2 The April trough and the Ramadan–Eid period

The estimated April reduction of 32.4% to 41.6% across all three programs, after adjustment for trend, is to our knowledge the first quantified estimate of a Ramadan-period reduction in cancer screening activity in Saudi Arabia. The finding is compatible with published evidence that elective healthcare utilization shifts in timing and intensity during Ramadan in Muslim-majority countries (20).

Several cautions apply to its interpretation. First, Ramadan spans two calendar months and did so in different proportions in the two study years, so a monthly series cannot localize the effect precisely; our sensitivity analyses (**Table 5**) show that the estimate’s magnitude, though not its direction or significance, depends on how exposure is defined. Second, the design cannot establish the causal nature of the association, nor can it separate fasting itself from the several co-occurring mechanisms that would produce the same monthly pattern: the Eid al-Fitr holiday closure of elective services, reduced clinic operating hours, staff leave, and patient preference for deferral. For this reason we describe the phenomenon as a Ramadan–Eid period effect rather than a Ramadan effect. Third, whether the trough represents cancellation of screening intent or temporal displacement into May cannot be determined from monthly aggregate counts; the May recovery visible in **Figure 1** is consistent with displacement, but consistency is not demonstration. This distinction matters programmatically: under displacement, the annual total is less affected than the monthly distribution suggests and the implication concerns scheduling capacity rather than net access.

With those caveats, the April trough is a calendar-predictable feature that screening managers can anticipate. Plausible operational responses include pre-Ramadan reminder and appointment campaigns, deliberate over-scheduling in the month following Eid to absorb any displaced demand, and avoidance of major campaign launches during the Ramadan–Eid window.

### 4.3 The Q4–2024 CRC deviation

The Q4–2024 CRC step change is statistically significant (p = 0.006) and cancer-specific, with no comparable signal in the breast or cervical programs. It represents a 46.6% excess (95% CI 13.1 to 89.9) above the trajectory predicted by the fitted secular trend, concentrated in November (+173% relative to November 2023) and December (+358% relative to December 2023), and it is invariant to the specification of the Ramadan term (**Table 5**). It is therefore a genuine departure from the preceding trend rather than an artifact of model specification.

What produced it cannot be determined from these data. The mechanism is unidentifiable from aggregate monthly counts, and several distinct explanations are compatible with the observed pattern:

- a targeted, time-limited national CRC screening campaign;
- a change in CHI reimbursement, coverage, or prior-authorization criteria taking effect in the final quarter;
- a provider-directed incentive or performance program;
- a change in procedure or diagnosis coding, or in how screening events are classified for reporting;
- year-end catch-up of deferred patient demand, or of provider claims submission, ahead of a benefit-year boundary;
- retrospective record entry, batch data submission, or another reporting-timing artifact.

We draw particular attention to the year-end claims-submission and reporting-timing explanations, which are administrative rather than clinical and would produce precisely the observed December peak without any change in underlying screening activity. The cancer-specificity of the signal argues against a general health-system capacity expansion, which would be expected to affect all three programs, but cancer-specificity is equally consistent with a CRC-specific coding or reimbursement change as with a CRC-specific clinical campaign.

External validation is therefore necessary. Administrative confirmation from CHI or the Ministry of Health regarding the timing and nature of any programmatic, reimbursement, or reporting change in late 2024 is required before this deviation can be attributed to any particular cause. Conditional on such confirmation identifying a campaign or incentive program, the implication would be that short-duration targeted CRC drives can produce substantial measurable increases in utilization among insured populations — a policy-relevant conclusion for scale-up planning, but one that cannot be drawn on the present evidence.

### 4.4 Male predominance in CRC screening counts

CRC screening counts were consistently higher among male than female beneficiaries (M:F count ratio 1.53 in 2023, 1.57 in 2024; 1.79 and 1.91 at ages 45–49). The leading explanation for this pattern is compositional. The CHI framework historically covers private-sector employees, and male labor force participation in Saudi Arabia substantially exceeds female participation, which produces a structurally male-dominant insured population (15). A male-dominant count is the expected consequence of a male-dominant denominator, independent of any difference in screening behavior.

Whether this male predominance reflects a difference in screening uptake or the sex composition of the CHI-insured population cannot be determined from count data; sex- and age-specific enrollment denominators are required to distinguish the two. We therefore report this as a distributional feature of screening counts and draw no inference about access, barriers, or inequity. For the same reason, the wider ratio observed at ages 45–49 is reported descriptively and is not interpreted as evidence of a pathway-entry barrier: the age–sex composition of the insured population at those ages is unknown to us and may itself differ from that at older ages.

Female and male CRC screening counts grew at similar relative rates between 2023 and 2024 (female +93.6%, male +98.2%), and the M:F count ratio changed only marginally, from 1.53 to 1.57. Whether this reflects any difference in relative uptake between the sexes cannot be determined without sex-specific eligible denominators.

Should denominator-based analysis subsequently establish that a genuine difference in screening rates exists, mechanisms worth examining would include differential referral in primary care, consistent with evidence of provider-level variability in screening practice (10). Targeted outreach to female beneficiaries at CRC screening entry age would be a reasonable programmatic response, but that recommendation is conditional on the denominator work being done first and is not supported by the present analysis alone.

### 4.5 Limitations

Several limitations must be acknowledged, of which the first is fundamental to the interpretation of everything reported here.

#### 4.5.1 Absence of denominators

The CHI datasets report screening event counts only. No eligible-population denominators stratified by age, sex, year, or program are published, and none were available to the author. Consequently no participation rate, coverage level, or standardized uptake ratio could be computed; no comparison with international benchmarks is possible; and no assessment of equity can be made. All findings describe absolute utilization counts.

#### 4.5.2 Growth in the insured population

CHI enrollment expanded over the period covered by these data (15). Count growth therefore conflates increased utilization with a growing denominator, and the two cannot be separated without enrollment files.

#### 4.5.3 Sex composition

As set out in §4.4, the male predominance in CRC counts cannot be distinguished from the sex composition of the insured population.

#### 4.5.4 Alternative explanations for the Q4–2024 CRC deviation

As set out in §4.3, coding changes, claims-submission or record-entry timing, and other administrative or reporting artifacts remain live explanations that these data cannot exclude.

#### 4.5.5 Ramadan exposure definition

Ramadan spans two calendar months, in different proportions in 2023 and 2024. Monthly aggregation cannot localize the effect, and the magnitude of the estimate varies with the exposure definition adopted (**Table 5**), although its direction and significance do not.

#### 4.5.6 Statistical power and model choice

The regressions rest on n = 24 monthly observations per cancer type with four parameters and 20 residual degrees of freedom. Inferences are indicative and require replication with longer series. OLS on log-transformed counts was used; negative binomial regression would be preferred with larger samples or individual-level data. The post-October 2024 indicator was coded for October through December; if the true onset was November, the estimate conflates two distinct periods and may understate the November–December magnitude.

#### 4.5.7 Coverage and generalizability

The datasets cover only CHI-insured beneficiaries and do not capture Ministry of Health–covered or uninsured individuals. No information on repeat screening, screening interval, test results, or diagnostic follow-up is available.

#### 4.5.8 Design

The study is observational and no causal attribution to any policy, campaign, or intervention is warranted.

### 4.6 Future directions

Extending the series to three or more years would increase power for trend analysis and permit detection of acceleration or deceleration. The highest-priority extension, however, is linkage of CHI screening counts to CHI enrollment denominators stratified by age, sex, year, and region: this is the prerequisite for any coverage estimate, for any comparison with international benchmarks, and for any assessment of whether the male predominance in CRC counts reflects a genuine difference in screening rates. Only if such analysis establishes a rate difference would qualitative or survey investigation of barriers facing female beneficiaries be warranted. Further linkage to the Saudi Cancer Registry would enable detection yield estimation. A formal pre-post or difference-in-differences analysis of the Q4–2024 CRC deviation, incorporating administrative confirmation of any campaign, reimbursement, or reporting change, would be required to establish its cause. Finally, resolving whether screening deferred during the Ramadan–Eid period is recovered in subsequent months or permanently lost would determine whether the April trough represents a scheduling challenge or a net access deficit.

## 5 Conclusion

This study provides the first national longitudinal description of cancer screening utilization among CHI-insured beneficiaries in Saudi Arabia, combining descriptive analysis with log-linear regression over 24 monthly observations across the breast, cervical, and colorectal programs. Using the adjusted primary model, statistically significant upward trends were identified for all three programs, with monthly increases of +3.0% (95% CI 1.5 to 4.5) for breast, +2.7% (95% CI 1.6 to 3.8) for cervical, and +4.6% (95% CI 3.3 to 5.9) for CRC screening, all p < 0.001. The April trough was quantified as a 32.4% to 41.6% reduction below model-predicted values, robust in direction and significance across four alternative Ramadan exposure definitions, providing a calendar-predictable basis for scheduling. The Q4–2024 CRC deviation was statistically distinguishable from the secular trend (+46.6%, 95% CI 13.1 to 89.9, p = 0.006) and cancer-specific, identifying a departure from trend whose mechanism requires administrative confirmation and which may be programmatic or administrative in origin. A persistent male predominance in CRC screening counts was documented but cannot be interpreted as inequity without sex-specific denominators.

Together these findings show that insurance-linked cancer screening volumes in Saudi Arabia rose measurably between 2023 and 2024, and that calendar effects and distributional differences are quantifiable features of that rise. They also delineate the boundary of what administrative count data can establish: converting screening volumes into meaningful statements about population coverage and equity requires enrollment denominators, registry linkage, and longer time series. Building that data infrastructure is the necessary next step.

## Data Availability

All data used in this study are publicly available through the Saudi open data portal at https://open.data.gov.sa/en/datasets. The specific datasets are: “Number of CHI Female Beneficiaries who Underwent Breast Cancer Screening by Age Group and Month in 2023G,” “Number of CHI Female Beneficiaries who Underwent Breast Cancer Screening by Age Group and Month in 2024G,” “Number of CHI Female Beneficiaries who Underwent Cervical Cancer Screening by Age Group and Month in 2023G,” “Number of CHI Female Beneficiaries who Underwent Cervical Cancer Screening by Age Group and Month in 2024G,” “Number of CHI Beneficiaries who Underwent Colorectal Cancer Screening by Age Group and Month in 2023G,” and “Number of CHI Beneficiaries who Underwent Colorectal Cancer Screening by Age Group and Month in 2024G.” The monthly counts analyzed are reproduced in full in **Table 4**, and the analysis script reproducing every coefficient reported in **Tables 2** and **5** from those counts is provided as **Supplementary File S1** and is also available from the author at alloghbi@kku.edu.sa.
